# Notification of Unexpected, Violent and Traumatic Death: A Systematic Review

**DOI:** 10.3389/fpsyg.2020.02229

**Published:** 2020-09-24

**Authors:** Diego De Leo, Josephine Zammarrelli, Andrea Viecelli Giannotti, Stefania Donna, Simone Bertini, Anna Santini, Cristina Anile

**Affiliations:** ^1^Australian Institute for Suicide Research and Prevention, Griffith University, Brisbane, QLD, Australia; ^2^Slovene Center for Suicide Research, Primorska University, Koper, Slovenia; ^3^De Leo Fund, Padua, Italy

**Keywords:** death notification, notifiers, recipients, emotional reaction, training, traumatic death, breaking bad news, receiving communication of death

## Abstract

**Background:** The way the death of a person is communicated can have a profound impact on the bereavement process. The words and expressions that are used to give the tragic news, the characteristics of who communicates it, the physical setting in which the notification is given, the means used (e.g., in person, via phone call, etc.) are just some of the factors that can influence the way survivors face one of the most difficult moments in their lives.

**Aim:** To review the literature on the topic of death notification to verify the state of the art related to this important procedure.

**Methods:** A systematic review was conducted with PRISMA criteria on English-written materials produced from 1966 to 2019.

**Results:** Out of the initial 3,166 titles considered, 60 articles were extracted for this review. A content analysis has revealed four main areas of interest: (1) protocols and guidelines; (2) emotional reactions of recipients and notifiers; (3) professional figures involved in the notification process; and, (4) types of death.

**Discussion:** The communication of death represents a complex and stressful experience not only for those who receive it but also for those who give it. Alongside the acquisition of a necessary technique and execution methods, the process should involve the selection of notifiers based on personality characteristics and communication styles.

**Conclusion:** Indications for the need of better training and protocols sensitive to different circumstances emerge. Adequate preparation can positively influence the quality of communication and the effects it produces, both on recipients and notifiers. In vocational training, more space should be devoted to this demanding task.

## Introduction

One of the crucial aspects related to the experience of death is the way in which an individual is informed of the loss of a loved one. People who die from sudden and violent causes (such as road accidents, murder, overdose, suicide, accidents at work, natural disasters, terrorist acts, etc.) often have partner, family and friends to whom the events that have led to death must be communicated (Adamowski et al., 1993; Stewart, 1999; Marco and Wetzel, 2012). But “death notification” is a significant moment that could change the life of survivors (those who suffer the loss) forever (Stewart, 1999). The words and expressions that are used to give the tragic news, the characteristics of who communicates it (doctor, policeman, nurse), the physical setting in which the notification is given (home, hospital, patrol car, offices of the police, etc.), the means used (in person, via phone calls, telegram, mail, or instant messaging) are just some of the factors that can influence the way survivors face one of the most difficult moments in their lives (Wheeler, 1994; Stewart, 1999). These circumstances represent painful memories related to the loss that will never be forgotten, and constitute a real break in the narrative story of the person, who will have to reconstruct new meanings around the loss and oneself (Stewart, 1999; Janzen et al., 2003-2004). In fact, most people remember—even after many years—every derelated to this painful communication. It would therefore be desirable that the notification of death could take place under the most appropriate conditions (Smith-Cumberland, 1994, 2007), as it potentially influences the path of elaboration of the mourning of the people involved. In this regard, it has been seen that people who cannot find any kind of meaning in their traumatic experience are more likely to develop persistent psychological distress, psychosomatic disorders, post-traumatic stress disorder (PTSD), and complicated bereavement (Horowitz et al., 1997; Murphy et al., 2002; Neimeyer et al., 2002; Armour, 2003).

De Leo Fund is a non-governmental organization (NGO) that deals with providing psychological support to people who have experienced an unexpected and violent loss (e.g., suicide, road accident, natural disaster, etc.). The people who turn to the NGO often report—together with the description of the experiences related to the loss—the details relating to the moment in which they were told of the death. The peculiarities of that sad notification frequently appear of remarkable importance in the narratives of clients of De Leo Fund, seemingly able to influence their bereavement process. In staff members of the NGO, this has promoted an interest in examining dynamics and variables that occur in the death notification process, by starting with a review of the existing literature on the subject. The main purpose of this study was, therefore, to examine the state of the art of the literature on this topic, with particular attention to critical issues and good practices that should be taken into account in the communication of a traumatic death, in order to eventually provide competent training to operators and improve the notification task.

Among the many issues to clarify are, for example, the identification of the figures most commonly involved in the notification process (e.g., who is responsible for communicating the death? To whom is a death communicated?), and the management of emotional reactions that may involve both the notifier and the target person (recipient) during the notification process, as well as the possible psychopathological consequences associated with the death notification experience. A further question concerns the general recognition in the literature of this area of research, and the current state of the art regarding existing protocols, guidelines, and practical recommendations. Ultimately, the research question from which our systematic review started was to investigate whether there are more adequate strategies or behaviors than others to be considered when it is required to make a death communication to survivors who they have lost a loved one for traumatic, unexpected, and violent death (i.e., an external cause of death: accident, suicide, homicide) and to understand if this process could have consequences on the physical and emotional level of those who are required to communicate and who those who receive the news.

## Methods

The compilation of this review followed PRISMA criteria. All the selected studies were examined, while no selection criteria were placed with respect to outcome and design of the individual studies.

There was no review protocol. Articles published in English language from 1966 to 2019 concerning the subject of death notification were selected. All articles that appeared without abstracts, or in the form of editorials or articles in periodicals, book chapters, book reviews or book chapters, dissertations and comments were excluded from the search.

The review of the literature focused on the notification of death in cases of unexpected, traumatic, and violent death. This refers to external causes of death only (i.e., accidents, suicides, and homicides). In light of this, we included only the studies investigating:

**A**. The characteristics of effective communication of traumatic death (conceptual articles reporting guidelines, protocols, and good practices);
**B**. Specific aspects of the death report, or seeking to improve the death notification process (e.g., articles assessing the effectiveness of new protocols; communication skills of the notifier, non-verbal language, gestures; needs of training on death notification of specific professional figures (for example, policemen);
**C**. Possible correlation between death notification and development of psychopathology (i.e., how the level of support perceived during the death notification process could correlate with psychopathological disorders);
**D**. Risk factors of the person to whom the notification is made; these have to be taken into account at the time of reporting death (e.g., how to communicate the death of a loved one to a drug abuser or a psychotic person);
**E**. Implications for the notifier (e.g., possible acute stress disorder for those who provide first aid; reactions of medical doctors/psychologists/other health professionals facing the news of the death of a patient);
**F**. Immediate and/or long-term reactions of those who receive the notification of a traumatic death.
  Defining the pertinent criteria was functional in differentiating the collected material and dividing it into more specific areas of investigation. Articles specifically investigating non-traumatic and non-violent death notification were excluded from this research. Deaths associated to palliative care were also excluded from the investigation. Exclusion also concerned:
**G**. Articles investigating perinatal death and infant death syndrome (SIDS);
**H**. Articles in which the death notification focused only on cases of cancer, cardiovascular and neurological diseases (these are not external causes of death).

Articles were identified through the following databases: Ebsco PsycINFO, Ebsco CINAHL, Scopus, Web of Science, MEDLINE PubMed, using as keywords: “death notification,” “death communication,” “notification of death,” “communication of death,” notification and “traumatic death,” communication and “traumatic death,” notification and “sudden death,” communication and “udden death.”

Researchers (JZ, AVG, SB, SD) carried out independently the bibliographic search for each keyword, as well as the subsequent elimination of duplicates. Each researcher (JZ, AVG, SB, SD) carried out searches in all databases. In checking for duplicates between the different search engines, the citations with slightly different title and the same abstract, the citations with the same title and abstract but different year of publication, and the citations with the same title and abstract but different title of the magazine were eliminated.

**Figure 1:**
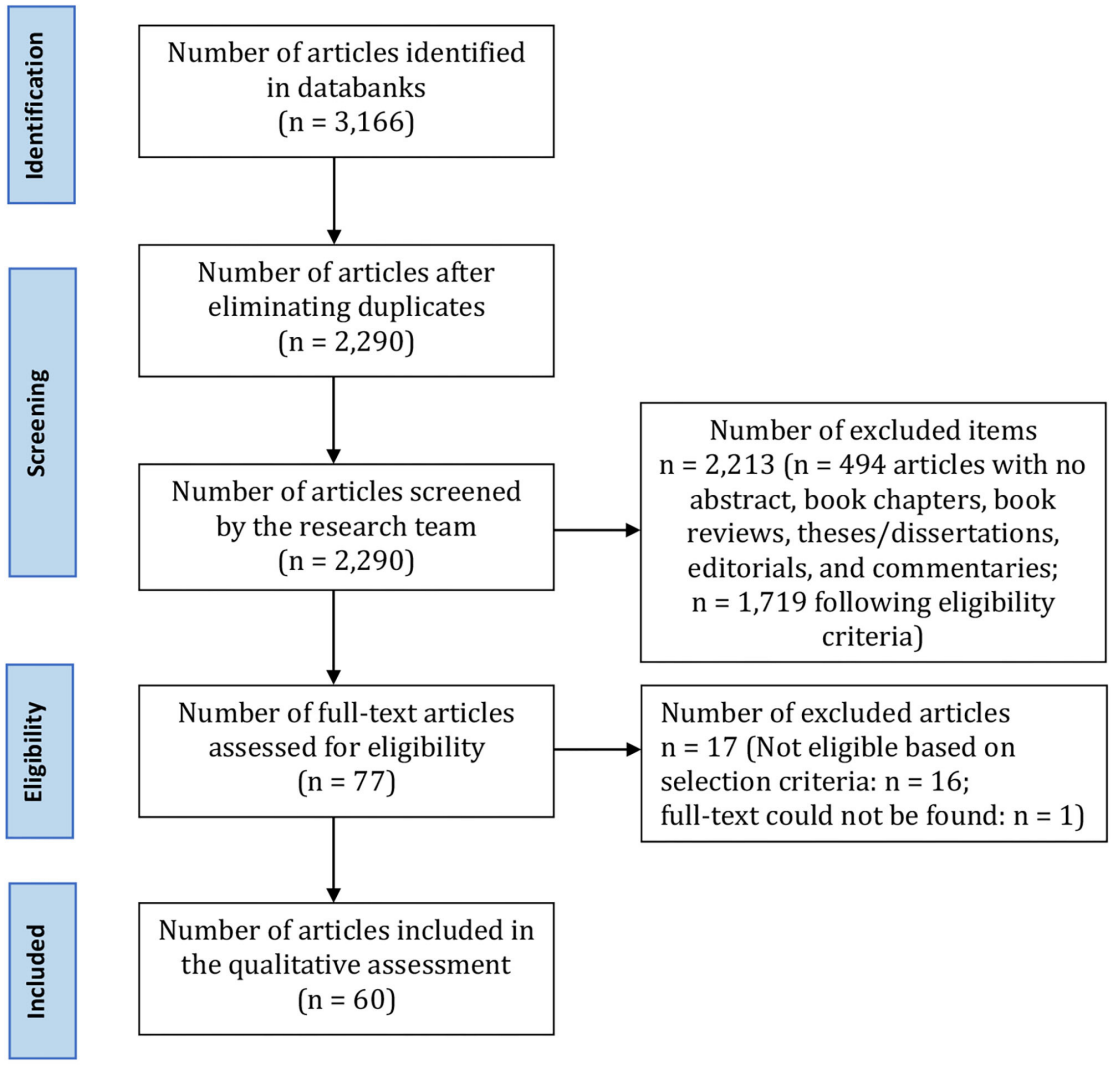
Decision tree for studies selection based on PRISMA criteria.

Once the final number of citations was obtained, three independent components of the research team (JZ, AVG, SB) carried out inclusion assessment in a standardized open mode. Disagreements between researchers were resolved by consensus methods. During the screening phase, it was not considered necessary to examine the full text of all articles. A fourth researcher (SD) complemented the reviewing process, while the senior author (DDL) oversaw the entire review process. Figure 1 shows the process of documents identification according to the PRISMA flowchart.

The assessment of risk of bias was performed at the study level. The main risk of bias in the inclusion criteria was related to the decision, implemented before conducting the content analysis, to include studies in which the type of death was not specified and studies that evaluated the overall death notification in relation to traumatic deaths and other types of death. In addition, studies with different size and methodology were also considered, resulting in a strong heterogeneity of the results. The risk of bias associated with the choice of the English language concerned the possibility that this language is associated with studies that are published faster and cited more often.

Figure 1 shows the process of identifying documents according to the PRISMA flowchart.

## Results

A total of 60 studies were included in our review. The analysis of study contents evidenced a remarkable degree of similarity, with most studies being of narrative type and conceptual content (i.e., most studies aimed at providing a global picture of the many aspects involved in the notification process). Despite the overlapping, we identified four dimensions as representative of the main aspects emerging from the studies. With the term “dimensions,” we want to indicate the main themes that emerged from the content analysis phase, i.e., summaries of information relating to a particular topic or domain of data with shared meanings. In particular, the identification of dimensions followed a reflexive, deductive thematic analysis phase. Once the data collection was complete, four researchers of the team (JZ, AVG, SD, SB) performed individually the analysis phases, during which they took notes on their initial impressions of each article. In a second moment, the contents of interest (i.e., those in line with the research question) were assigned labels (a few words or a short sentence), which had the purpose of clearly evoking the relevant characteristics of the papers, in order to be able to encode them. Then, researchers—with full agreement between them—defined a list of themes, which ended in four dimensions that guided the subsequent research phases. The dimensions identified are: (1) protocols and guidelines (number of studies = 51); (2) emotional reactions of the recipients and those who communicate the news, and/or pathologies that can influence the bereavement process (*n* = 46); (3) professional figures who perform the notification and recipients of the bad news (*n* = 59); (4) type of death to be communicated (*n* = 56) (See Table 1).

**Table 1:** Dimensions identified through the reviewing process.

**1. Protocols, guidelines, educational programs, communication**
strategies and techniques, role-playing: *n* = 51
General protocols/guidelines: *n* = 20
Training programs/exercises/guidelines for emergency department: *n* = 7
Specific protocols (e.g., GRIEV_ING, SPIKES, ABCDE, Dryer, “In Person, In Time,” DNR): *n* = 13
Role play/simulations: *n* = 4
Police officer procedures: *n* = 2
Privacy management processes in the notification process: *n* = 1
Telephone communication about death: *n* = 1
Practical recommendations for schools and school professionals: *n* = 1
Bibliotherapy for children: *n* = 1
Strategies/techniques for communicating death in hospices and nursing homes: *n* = 1
**2. Emotional reactions of notifiers and recipients and**
pathologies/circumstances that can influence the mourning process:
*n* = 46
Emotional reactions of both the communicator and the recipient: *n* = 19
Emotional reactions of those who communicate: *n* = 12
Emotional reactions of recipients: *n* = 15
**3. Professional figures involved in the notification process: *n* = 59**
Doctors: *n* = 7
Healthcare workers: *n* = 18
General/unspecified category: *n* = 6
Multidisciplinary team (medical and nursing staff, psychologists, priests/chaplains. social workers, etc.):
*n* = 15
Law enforcement: *n* = 3
Students: *n* = 2
Trainees: *n* = 3
Nurses: *n* = 3
School workers: *n* = 1
Disability service personnel: *n* = 1
**4. Type of death that should be notified: *n* = 56**
Traumatic death (e.g., road accident, homicide, overdose, suicide): *n* = 9
Unexpected and sudden death: *n* = 20
Type of death not specified: *n* = 10
Miscellaneous types of death (traumatic, unexpected/sudden, due to illness, natural): *n* = 17

*Components of identified dimensions*.

As anticipated, the total number of studies from each area exceeds the total number of studies selected due to the overlap of multiple thematic areas within the same study.

Death notification in relation to the first identified dimension concerns the state of the art regarding theories and practical training implemented in communicating death. Here, heterogeneity of collected data and difficulty in distinguishing protocols and guidelines from training programs and simulation exercises should be underlined.

The second area/dimension concerns the role of the emotions of those who communicate and receive the notification of death, and the risk factors/circumstances that can influence the bereavement process (psychopathologies, disabilities, people at risk, etc.).

Nurses, doctors, paramedics, social workers, psychologists, and policemen are placed in the third group of studies as main actors involved in the notification process. Characteristics of the population receiving the notification are also reported in this area.

In relation to the type of death, some studies have addressed the issue of death notification in relation to traumatic deaths, while others have also dealt with natural deaths, or have not specified the type of death or have treated sudden and unexpected death without specifying their cause.

Overall, the evaluation of the 60 studies included in our review revealed the presence of a vast heterogeneity between methodologies and research designs. The typology of the studies appeared distributed as follows (Table 2): conceptual/narrative studies (*n* = 24), surveys (*n* = 9), cross-sectional surveys (*n* = 2), reviews (*n* = 3), qualitative studies (*n* = 8), pre-post design studies (*n* = 7), validation studies (*n* = 4), prospective observational (*n* = 2), mixed-method design study (qualitative/quantitative) (*n*= 1). The prevalence of conceptual/narrative studies within our review and the scarcity of quantitative studies did not permit to aggregate data and provide numbers resulting from contamination. It was not possible to trace the risk of bias of each individual study and carry out an assessment of the outcome and results of each individual work. From our review of the literature we tried to obtain a picture of the state of art of a topic we consider of importance, such as the problem of death communication in the event of an unexpected, traumatic, and violent death.

**Table 2:** Characteristics of studies included in the systematic review.

**Author (year)**	**Country**	**Target population**	**Sample (*n*)**	**Study design**	**Dimensions/themes**			
					**Protocols and guidelines**	**Emotional reactions and pathologies**	**Type of professionals**	**Types of death**
Dubin and Sarnoff (1986)	Pennsylvania (USA)	Doctors and health personnel of emergency dept.s	NA	Conceptual/ narrative	Protocols/ General guidelines	Emotional reactions of recipients	Health professionals	Sudden and unexpected death
Parrish et al. (1987)	Florida (USA)	Family members of patients who died in Emergency Department	66	Survey	NA	Emotional reactions of recipients	Health professionals	Sudden and unexpected death
Spencer et al. (1987)	California (USA)	Homicide detectives of Los Angeles Police Department	50 Ss filled in a questionnaire. 21 made also a telephone interview	Cross-sectional survey	Police officer procedures	Emotional reactions of notifiers and recipients	Law enforcement personnel	Sudden and unexpected death
Haglund et al. (1990)	Washington DC (USA)	Law enforcement, medical examiner and coroner officers	NA	Conceptual/ narrative	NA	Emotional reactions of notifiers and recipients	Multidisciplinary Team (law enforcement, medical examiner and coroner offices)	Miscellaneous
Schmidt et al. (1992)	Oregon (USA)	Residents of Oregon Health Sciences University	NA	Conceptual/ narrative	Role games/Simulations	NA	Health professionals	NA
Adamowski et al. (1993)	Canada	2 groups of survivors in Ottawa General Hospital.	66	Survey	Protocols/ General guidelines	Emotional reactions of notifiers and recipients	Multidisciplinary Team (doctors, nurses, psychologists, chaplains, social workers, etc.)	Miscellaneous
Swisher et al. (1993)	Pennsylvania (USA)	45 resident and 20 physicians in emergency dept.s at the Medical College of Pennsylvania	65	Survey	Protocols/General guidelines	Emotional reactions of notifiers	Health professionals	Sudden and unexpected death
Marrow (1996)	UK	Emergency dept.s personnel	NA	Conceptual/ narrative	Protocols/ General guidelines	Emotional reactions of notifiers and recipients	Health professionals	Sudden and unexpected death
Moroni Leash (1996)	California (USA)	Medical professionals, University students in death and dying classes and family members of newly admitted intensive care unit patients.	200 medical professionals, 100 University students, 100 family members.	Conceptual/narrative	Specific Protocols (e.g., SPIKES, ABCDE, “In Person, In Time,” DNR, etc.)	NA	Health professionals	Miscellaneous
Viswanathan (1996)	New York (USA)	Physicians in the departments of medicine, surgery, family practice, and psychiatry at the State University of New York Health Science Center at Brooklyn	155	Survey	NA	Emotional reactions of notifiers	Doctors	Sudden and unexpected death
Von Bloch (1996)	Texas (USA)	Health care professionals	NA	Conceptual/ narrative	Protocols/ General guidelines	Emotional reactions of notifiers and recipients	Health professionals	Sudden and unexpected death
Ahrens and Hart (1997)	Illinois (USA)	General emergency physicians	122	Survey	Training programs for emergency department health workers (EDECT e CME)	Emotional reactions of notifiers	Doctors	Sudden and unexpected death
Olsen et al. (1998)	Chicago (USA)	Emergency dept.s personnel	NA	Conceptual/ narrative	Protocols/ General guidelines	Emotional reactions of notifiers and recipients	Multidisciplinary Team (doctors, nurses, psychologists, chaplains, social workers, etc.)	Sudden and unexpected death
Smith et al. (1999)	Maryland (USA)	Emergency physicians, paramedics and other emergency personnel	NA	Conceptual/narrative	Training programs for emergency department health workers (EDECT e CME)	Emotional reactions of notifiers	Health professionals	Sudden and unexpected death
Stewart (1999)	Florida (USA)	Those involved in notifying a road accident-related death	NA	Conceptual/narrative	Protocols/General guidelines	Emotional reactions of notifiers and recipients	Multidisciplinary Team (doctors, nurses, psychologists, chaplains, social workers, etc.)	Traumatic death
Stewart et al. (2000)	Florida (USA)	Participants from 14 different professions potentially involved in notifying death	240	Survey	NA	Emotional reactions of notifiers and recipients	Multidisciplinary Team (doctors, nurses, psychologists, priests/ chaplains, social workers, etc.)	Miscellaneous
Kaul (2001)	Michigan (USA)	Emergency physicians, paramedics and other emergency personnel	NA	Conceptual/narrative	Specific Protocols (e.g., SPIKES, ABCDE, “In Person, In Time,” etc.)	Emotional reactions of recipients	Multidisciplinary Team (doctors, nurses, psychologists, chaplains, social workers, etc.)	Miscellaneous
Stewart et al. (2001)	Florida (USA)	Death notifiers (law enforcement officers, emergency medical technicians, victim advocates, coroners, etc.) in 7 cities of USA.	245	Survey	Protocols/ General guidelines	NA	Multidisciplinary Team (doctors, nurses, psychologists, priests/ chaplains, social workers, etc.)	Miscellaneous
Benenson and Pollack (2003)	England	Emergency medicine residents	70	Prospective observational	Training programs for emergency department health workers (EDECT e CME)	Emotional reactions of notifiers	Doctors	Sudden and unexpected death
Janzen et al. (2003-2004)	Canada	Parents who had experienced the sudden death of a child in Ontario	20	Qualitative study	Protocols/General guidelines	Emotional reactions of notifiers and recipients	Multidisciplinary Team (doctors, nurses, psychologists, priests/ chaplains, social workers, etc.)	Miscellaneous
Servaty-Seib et al. (2003)	Indiana (USA)	School communities	NA	Conceptual/ narrative	Practical recommendations for schools and school professionals	Emotional reactions of recipients	School workers	Miscellaneous
Hart and DeBernardo (2004)	Baltimore (USA)	Law enforcement personnel	NA	Conceptual/ narrative	Protocols/ General guidelines	Emotional reactions of notifiers and recipients	Law enforcement personnel	Miscellaneous
Levetown (2004)	Texas (USA)	Emergency care personnel	NA	Conceptual/ narrative	Training programs for emergency department health workers (EDECT e CME)	Emotional reactions of recipients	General category/ unspecified	Miscellaneous
Deffner and Bell (2005)	Arizona (USA)	Nurses in state of Arizona	190	Survey	NA	Emotional reactions of notifiers	Nurses	Unspecified type of death
Goodrum (2005)	North Carolina (USA)	Bereaved of murdered loved ones	32	Qualitative study	NA	Emotional reactions of recipients	General category/ unspecified	Traumatic death
Hobgood et al. (2005)	North Carolina (USA)	Residents in emergency medicine	20	Pre-post study	Training programs for emergency department health workers (EDECT e CME);	Emotional reactions of notifiers	Trainees	Sudden and unexpected death
Smith-Cumberland and Feldman (2005)	Maryland, Pennsylvania and Utah (USA)	Emergency medical technicians from 14 states.	136	Survey	Protocols/General guidelines	Emotional reactions of notifiers	Doctors	Unspecified type of death
Eberwein (2006)	Maryland (USA)	Mental health professionals	NA	Conceptual/ narrative	Protocols/ General Guidelines	Emotional reactions of recipients	Health professionals	Traumatic death
Quest et al. (2006)	Georgia (USA)	Undergraduate medical students on their fourth-year.	37	Prospective observational	NA	NA	Students	Sudden and unexpected death
Smith-Cumberland and Feldman (2006)	Wisconsin (USA)	Emergency medical professionals	83	Pre-post study	Training programs for emergency department health workers (EDECT e CME)	NA	Health professionals	NA
Scott (2007)	England	Emergency medical professionals and police officers	NA	Conceptual/narrative	Protocols/ General guidelines	Emotional reactions of notifiers and recipients	Multidisciplinary Team (doctors, nurses, psychologists, chaplains, social workers, etc.)	Traumatic death
Miller (2008)	Florida (USA)	Professionals potentially involved in notifying death	NA	Conceptual/narrative	Protocols/General guidelines	NA	General category/unspecified	Traumatic death
Mitchell (2008)	Maryland (USA)	Professionals potentially involved in notifying death	NA	Conceptual/narrative	Protocols/General guidelines	Emotional reactions of recipients	General category/ unspecified	NA
Hobgood et al. (2009)	North Carolina (USA)	Fourth-year medical students at the University of North Carolina	138	Pre-post study	Specific Protocols (e.g., GRIEV_ING, SPIKES, ABCDE, “In Person, In Time,” DNR, etc.)	NA	Students	Unspecified type of death
Parris (2011)	UK	Emergency medical professionals	NA	Conceptual/narrative	Protocols/General guidelines	Emotional reactions of recipients	Health professionals	NA
Douglas et al. (2012)	Canada	Paramedics in urban and rural areas of Ontario	28	Qualitative study	Role games/simulations	Emotional reactions of notifiers and recipients	Health professionals	Miscellaneous
Marco and Wetzel (2012)	Ohio (USA)	Patients who were involved in a fatal motor vehicle crash between 2005 and 2009.	21	Cross-sectional survey study	Protocols /General Guidelines	Emotional reactions of recipients	General category/unspecified	Traumatic death
Roe (2012)	Michigan (USA)	Emergency medical professionals	NA	Conceptual/narrative	Protocols/General guidelines	NA	Multidisciplinary Team (doctors, nurses, psychologists, chaplains, social workers, etc.)	Sudden and unexpected death
Shaw et al. (2012)	Australia	Doctors employed in Sydney metropolitan hospitals.	31	Mixed-method design (quantitative/ qualitative)	Role playing/simulations	NA	Doctors	Sudden and unexpected death
Douglas et al. (2013)	Canada	Paramedics in urban and rural areas of Ontario	28	Qualitative study	NA	Emotional reactions of notifiers	Health Professionals	Miscellaneous
Hobgood et al. (2013)	North Carolina (USA)	Emergency medical service personnel	30	Pre-post design	Specific Protocols (e.g., GRIEV_ING, SPIKES, ABCDE, “In Person, In Time,” DNR, etc.)	NA	Health Professionals	Unspecified type of death
Scott (2013)	England	Emergency medical professionals	NA	Conceptual/narrative	Training programs for emergency department health workers (EDECT e CME)	NA	Health Professionals	Sudden and unexpected death
Shoenberger et al. (2013)	South California (USA)	Physicians of emergency departments	NA	Review article	Specific Protocols (e.g., GRIEV_ING, SPIKES, ABCDE, “In Person, In Time,” etc.)	Emotional reactions of notifiers and recipients	Doctors	Miscellaneous
Shomoossi et al. (2013)	Iran	Nurses working in hospitals in Sabzevar, in Iran.	97	Development and validation of a scale	Specific Protocols (e.g., SPIKES, ABCDE, “In Person, In Time,” etc.)	Emotional reactions of notifiers and recipients	Nurses	Miscellaneous
Sobczak (2013)	Poland	Doctors involved in death notification	NA	Conceptual/ narrative	Specific Protocols (e.g., SPIKES, ABCDE, “In Person, In Time,” etc.)	NA	Doctors	Unspecified type of death
Galbraith et al. (2014)	Midwestern (USA)	Senior-level nursing and social work students	32	Development of a valid simulation model	Role games/simulations	Emotional reactions of notifiers and recipients	Multidisciplinary Team (doctors, nurses, psychologists, social workers, etc.)	Sudden and unexpected death
Rivolta et al. (2014)	Torino (Italia)	Health care nurses of two nursing homes and two hospices	55	Qualitative study	Strategies for communicating death in hospices and nursing homes	NA	Nurses	Unspecified type of death
Baumann and Stark (2015)	New Jersey (USA)	Forensic death investigators and other death notifiers	NA	Conceptual/narrative	Protocols/ General guidelines	Emotional reactions of recipients	Multidisciplinary Team (doctors, nurses, psychologists, chaplains, social workers, etc.)	Traumatic death
Reed et al. (2015)	Ohio (USA)	First-year pediatric and internal medicine residents	44	Pre-post design	Specific Protocols (e.g., GRIEV_ING, SPIKES, ABCDE, “In Person, In Time,” etc.)	Emotional reactions of notifiers and recipients	Trainees	Unspecified type of death
Basinger et al. (2016)	Midwestern (USA)	College students who had lost a parent or a sibling.	21	Qualitative study	Privacy management processes in the notification of death (CPM)	Emotional reactions of recipients	NA	Miscellaneous
Carmack and DeGroot (2016)	Virginia (USA)	Lay people recruited via social media, and other means	302 in study 1; 319 in study 2)	Development and validation of a new scale	Specific Protocols (e.g., CADS, GRIEV_ING, SPIKES, ABCDE, “In Person, In Time,” etc.)	Emotional reactions of notifiers	Multidisciplinary Team (doctors, psychologists, etc.)	Unspecified type of death
Peters et al. (2016)	New South Wales (Australia)	Individuals bereaved by suicide	10	Qualitative study	NA	Emotional reactions of recipients	Multidisciplinary Team (doctors, nurses, psychologists, chaplains, social workers, etc.)	Traumatic death
Veilleux and Bilsky (2016)	Arkansas (USA)	Therapists and residents after a patient death (e.g., suicide)	NA	Conceptual /narrative	Protocols/General guidelines	Emotional reactions of notifiers	Multidisciplinary Team (doctors, nurses, psychologists, chaplains, social workers, etc.)	Traumatic death
Arruda-Colli et al. (2017)	Maryland (USA)	Storybooks about dying, death and bereavement in English, French or Spanish, published 1995-2015	210	Review article	Bibliotherapy for children	Emotional reactions of recipients	General category/ unspecified	Miscellaneous
Brand and Mahlke (2017)	Germany	German police officers	NA	Conceptual/ narrative	Specific Protocols (e.g., SPIKES, ABCDE, “In Person, In Time,” DNR, etc.); Police officer procedures	Emotional reactions of recipients	Law enforcement personnel	Sudden and unexpected death
Karam et al. (2017)	Lebanon	Residents of PGY3 and PGY4 Lebanese anesthesiology	16	Pre-post training	Specific Protocols (e.g., GRIEV_ING, SPIKES, ABCDE, “In Person, In Time,” DNR, etc.)	Emotional reactions of notifiers	Trainees	Miscellaneous
Ombres et al. (2017)	Maryland (USA)	Internal medicine residents	67	Review article	Telephone communication about death	Emotional reactions of notifiers and recipients	Health professionals	Sudden and unexpected death
Shakeri et al. (2017)	Chicago (USA)	American University - 4 years emergency medicine training program	40	Validation study	Specific Protocols (e.g., GRIEV_ING, SPIKES, ABCDE, “In Person, In Time,” DNR, etc.)	NA	Health professionals	Unspecified type of death
Tuffrey-Wijne and Rose (2017)	UK	Social care staff that worked in residences for intellectually disable people	20	Qualitative study	NA	Emotional reactions of notifiers and recipients	Disability service personnel	Unspecified type of death
Williams-Reade et al. (2018)	California (USA)	Pediatric surgery residents	15	Pre-post study	Specific Protocols (e.g., SPIKES, ABCDE, “In Person, In Time,” etc.)	Emotional reactions of notifiers and recipients	Health professionals	Sudden and unexpected death

### First Dimension: Protocols and Guidelines

In relation to the first dimension, a total of 51 studies concerning death notification have highlighted the current state of the art regarding intervention plans, guidelines, strategies and protocols to make an adequate communication (see Table 2). In this dimension, a total of 20 studies focused on the description of general protocols and general guidelines that can be used during the death notification process (Table 2).

A total of 7 studies focused on the description of training programs for emergency department health workers aimed at improving the communication skills of the professionals involved in the role of notifier (Table 2). Only one study highlighted the usefulness of a specific training on death education in critical emergency situations, called the “*Emergency Death Education and Crisis Training*” (EDECT) program. It is a theoretical-experiential training that proposes role-playing and group activities, aimed at modifying the attitudes with respect to the theme of death and most common behaviors of emergency doctors who must carry out death communication. The program also includes specific medical education hours on the notification process (CME) (Smith-Cumberland and Feldman, 2006).

A total of 13 studies have investigated some specific protocols that can be used as a reference during a death notification (Table 2). One of the most used is the GRIEV_ING protocol (Hobgood et al., 2009, 2013; Shoenberger et al., 2013; Reed et al., 2015; Carmack and DeGroot, 2016; Karam et al., 2017; Shakeri et al., 2017). In this regard, the importance of an educational intervention, based on simulation, is highlighted for the acquisition of the skills necessary to face a death notification. In the United States, one of the most popular protocols is the so-called “*Six-Step Protocol”* for the delivery of bad news (SPIKES: acronym for the words Setting, Perception, Invitation, Knowledge and Empathy). It is a protocol aimed primarily at doctors who provide information on unfavorable prognoses, but can also be used to inform the family of the death of a patient (Scott, 2013; Shomoossi et al., 2013; Williams-Reade et al., 2018). One study described the ABCDE notification strategies that propose accurate intervention plans for healthcare professionals who are required to make a death notification (Shomoossi et al., 2013). A study examined a further protocol, developed by Dyer (2001), in which the more general guidelines for the notification of death are expanded, adding rules relating to telephone communication and the timing appropriate for meeting with the person to whom the death news should be referred (Sobczak, 2013). A study focuses on the communication protocol “*In Person, In Time,”* which provides useful information to effectively reduce the stress level of the notifier at the time of communication (Sobczak, 2013). An article focused on a learning program, called the DNR approach (Brand and Mahlke, 2017): it is an educational program useful to facilitate learning, based on practical experiences within peer groups, to strengthen notification skills. Four studies have highlighted the usefulness of role-playing and immersive simulation experiences of the notification process (Schmidt et al., 1992; Douglas et al., 2012; Shaw et al., 2012; Galbraith et al., 2014). Two studies have shown notification procedures for law enforcement, in particular police officers, often involved in traumatic death experiences, such as traffic accidents, suicides and homicides (Spencer et al., 1987; Brand and Mahlke, 2017).

A study highlighted the importance of protecting privacy in the information provided to partner/family/friends during the notification of the death of a loved one (Basinger et al., 2016). Another study presented strategies on how to make an adequate death notification commencing by a phone call (Ombres et al., 2017). Whenever possible, phone calls should not be the only way to communicate the traumatic death of someone: direct contact with survivors should always be encouraged.

An article described practical recommendations for notification of death to students in case of a loss within the school environment (Servaty-Seib et al., 2003).

Another study highlighted the potential of bibliotherapy as a tool to facilitate the communication about death to children who need to face the loss of a loved one (Arruda-Colli et al., 2017). A study described some techniques and strategies for communicating the death of a resident in nursing homes and hospice-type structures (Rivolta et al., 2014).

### Second Dimension: Emotional Reactions of Notifiers and Recipients and Circumstances or Pathologies That Can Influence the Bereavement Process

A total of 46 studies (Table 2) have investigated death notification in relation to the main emotional experiences that accompany a death notification process, taking into account both the experience of the notifier and those who receive the news. A total of 19 studies (Table 2) described the notification process, taking into account both perspectives. From the notifier's point of view, the notification process is mostly judged as difficult and stressful (Adamowski et al., 1993; Stewart et al., 2000; Janzen et al., 2003-2004; Hart and DeBernardo, 2004; Douglas et al., 2012; Williams-Reade et al., 2018). The reactions most commonly described by the notifier are: anxiety, guilt, sadness, identification with the target, discomfort, avoidance, anguish, frustration, isolation, insomnia, lowering of mood, recurrent nightmares, feelings of helplessness, substance abuse, marital conflict, PTSD, chronic stress response syndrome, and professional burnout (Spencer et al., 1987; Veilleux and Bilsky, 2016; Tuffrey-Wijne and Rose, 2017). In general, professionals involved in communicating the death of a person highlighted a lack of sufficient preparation for carrying out the task and the need to acquire more skills for managing the emotional reactions of those who receive the bad news and own emotions (Olsen et al., 1998; Douglas et al., 2013). Considering the target's perspective, the notification task is more commonly associated with reactions such as: emotional trauma, pain, despair, anger, crying, screaming, sadness, aggression, depression, emotional distress, strengthening of significant relationship ties, perception of stigma, social isolation, avoidance of relationships, distress, sense of emptiness, increased heart rate, fainting, cardiac arrest, and nausea (Haglund et al., 1990; Adamowski et al., 1993; Janzen et al., 2003-2004; Hart and DeBernardo, 2004; Scott, 2007; Galbraith et al., 2014; Basinger et al., 2016).

Twelve studies dealt in particular with the notifier's emotional experience and a total of 15 studies (Table 2) examined the experience of recipients. With regards to the notification experience from the side of the notifier, some detailed information is provided regarding the long-term effects of stress and discomfort perceived during the process, as well as some of the most common psychopathological risk factors (Stewart, 1999; Hobgood et al., 2013). Finally, the studies that highlight the main reactions of those who receive a death notification underline the devastating impact that this experience, if not adequate, could have in the lives of recipients, as well as the significant influence of this experience on the bereavement process and on the onset of psychopathology and possible increase in suicidal risk. Sudden and violent deaths can trigger acute psychological responses; they can increase the risk of developing complicated bereavement, in addition to the onset of physical and psychological symptoms. In turn, these could lead to an increase in suicidal risk and general mortality (Kaul, 2001; Mitchell, 2008; Parris, 2011; Baumann and Stark, 2015; Peters et al., 2016; Brand and Mahlke, 2017; Ombres et al., 2017).

### Third Dimension: Professional Figures Involved in the Notification Process

Nearly all studies (*n* = 59) dealt with this aspect (Table 2). The review revealed significant diversity among operators involved in the death notification process. The professional category most involved in the role of notifier is that of healthcare workers (evidenced in 18 studies). In six studies, professional role of the notifier is unspecified. In others, several professional figures are seen as working synergistically—in communicating the death of a person—within a multidisciplinary team (*n* = 15). Among these are: medical and nursing staff, religious officers and priests, psychologists, social workers, etc.) (Table 2). A total of seven studies highlighted the role of doctors in the death notification task. Three studies focused on the role of law enforcement officers. Three articles focused on the figure of nurses; another study on that of school operator. Students are also the target of two studies as well as studies on trainees (*n* = 3). Finally, one article considered staff working in services for people with disabilities (see Table 2 for specific references).

### Fourth Dimension: Type of Death to Be Notified

When it comes to death notification, it is fundamental to dwell on the peculiar characteristics of each type of death to examine the components that may influence the different loss experiences. A death from natural causes has a very different impact on partner/family/friends than a death from sudden and violent causes. A total of 56 studies focused on providing information about the different types of death. Nine studies focused on traumatic death experiences (such as traffic accidents, suicides, murders, overdoses) (Table 2). Other studies have examined deaths both from sudden and unexpected causes (*n* = 20); in some studies the theme of death has been studied without specifying its type (*n* = 10); finally, a number of studies (*n* = 17) have considered a miscellaneous ensemble of deaths (traumatic, unexpected and sudden, by disease, natural) (Table 2).

## Discussion

In recognition of the importance of the notification process, protocols, and recommendations have been developed to help professionals and rescuers called to perform death notifications (Hall, 1982; Parrish et al., 1987; Collins, 1989; Wells, 1993; Williams and Frangesch, 1995; Byers, 1996; Ptacek and Eberhardt, 1996; Von Bloch, 1996; Spungen, 1998; Boss, 1999, 2002; Stewart, 1999; Benenson and Pollack, 2003; Miller, 2003a,b, 2004; Eberwein, 2006; Nardi and Keefe-Cooperman, 2006). It is important, in fact, that notifiers (doctors and other health workers, psychologists, priests, members of law enforcement, and the school community) do not underestimate the impact that they could have during the first meeting with the partner/family/friends of the deceased. Professionals who do not develop an awareness of how their attitudes can influence the notification process are likely to cause more stress in recipients, often in an unintended way, and to live the moment of communication with greater anxiety.

Ideally, the notifier should be well-informed about the details of the death, have enough time to support the survivors of the deceased, and be able to respond to the physical and emotional reactions exhibited by them (denial, anger, aggression, withdrawal, isolation, tears, pain, guilt, fear, etc.) (Wright, 1996). It is also important to consider the psychophysical characteristics of the individuals who are going to be informed, and evaluate the secondary health risks that may arise following the notification of death. In some cases, it may be useful to evaluate suicide risk or to take individual risk factors into account. For example, it often happens that people with intellectual disabilities are over-protected and often not informed of the death of a loved one (Tuffrey-Wijne and Rose, 2017).

What emerges from the literature is that not much information is available on how to deal with sudden death, and that there is still a shortage of educational material in this area for emergency professionals (Smith et al., 1999). In addition, standardized protocol for making death notifications by phone in different contexts appear to be lacking (Ombres et al., 2017). The professional figures mainly called to deal with the notification processes often do not receive adequate training to learn how to provide painful communications in the most appropriate way. This causes the notification process to be perceived as a particularly difficult and stressful event for notifiers, as well as for those who are required to receive the bad news. It is known, in fact, that this task emotionally involves not only the family members and other survivors of the deceased but also the professionals who are called to communicate death (Meunier et al., 2013). Moreover, communicating death to a close person or family member without an adequate strategic plan can contribute to aggravating the pain of loss (Mitchell, 2008; Ombres et al., 2017). Improving the notifiers' skills and competences, along with understanding the possible reactions of those who receive a death report, can increase the chances that the death notification process may result as sensitive, empathetic, respectful, and compassionate.

The importance of an intervention that aims to facilitate the bereavement process from a traumatic loss has been discussed in the literature by taking into consideration the contribution of different professional groups, including the clergy (Frantz et al., 1996; Weaver et al., 1996), health workers (Gyulay, 1989) and law enforcement (Clark, 1981). A multidisciplinary team engaged in the notification process can be made up of doctors, nurses, psychologists, social workers, policemen, priests and, in general, sensitive, professional and caring staff who act through a cooperative and coordinated approach (Walters and Tupin, 1991; Adamowski et al., 1993; Anderson, 1993). It is essential, in fact, that each team member is aware of their role and the complexity of the notification process, in order to promote and ensure effective support for survivors (Young et al., 2012; Groos and Shakesperare-Finch, 2013).

In this regard, the need for a deeper understanding of the problems that people face when losing a loved one has been well-highlighted, as well as the need for professionals to be better trained in the difficult task of death notification (Gyulay, 1989; Kalkofin, 1989; Neidig and Dalgas-Pelish, 1991; Weaver, 1993; Parry, 1994; Weaver et al., 1996). There is evidence that most staff have a desire to improve their notification skills for managing crisis situations, especially in emergency settings (Olsen et al., 1998).

Studies outlining protocols and guidelines have highlighted some aspects to be especially promoted in the notification process: simplicity, brevity, practicality, proximity, immediacy, and expectation of a reasonable result (Aguilera, 1998; Mitchell, 2004). Some studies have supported the preventive importance of a skilled and sensitive death notification, through a set of fundamental tasks and events, deemed salient regardless of who makes the notification and the setting in which it is carried out. To perform a “proper” death notification, the various components include: (a) correct identification of both the deceased person and the partner/family/friends to be notified; (b) a first contact (preferably not by telephone) with the survivors and an invitation to a meeting at their home or hospital; (c) to provide all the details about the accident and the medical procedures implemented (if any). It has been seen that both the physical setting in which information is given and the speed with which it is exposed can significantly influence the survivors' ability to assimilate all the details of communication; (d) to provide the news of death. In this regard, it is important to use clear and direct language and avoid the use of euphemisms, especially in dealing with children; (e) to respond to survivors' reactions by offering immediate emotional support; (f) to give the opportunity to view the body after the notification; (g) to offer short and long-term assistance through follow-up following the notification process, which guarantees survivors that they may obtain further information on death circumstances (this is particularly important for those types of losses that involve a high risk of developing PTSD) (Dubin and Sarnoff, 1986; Spencer et al., 1987; Cooke, 1993; Tye, 1993; Stewart, 1999; Stone et al., 1999; Kaul, 2001; Stewart et al., 2001; Li et al., 2002).

Several studies that examined the impact on recipients for the notification received revealed significantly worse results if death, and in particular the causes of death, were communicated by telephone. The limits of this approach—which should only be used in cases where survivors are really far away (Stewart, 1999)—have been repeatedly stressed. Nevertheless, telephone notification is still a widely used communication method, in particular by law enforcement officers when they are required to report deaths resulting from road accidents, homicides and/or suicides (Spencer et al., 1987; Stewart, 1999; Miller, 2008). In the particular case of deaths resulting from a murder, it would be appropriate to inform the deceased's partner or family members of the possibility that the media can contact them. It would be also useful to provide them with suggestions on how to manage various aspects, including those related to the protection of privacy (Clark, 2011; Baumann and Stark, 2015).

At least one study has tried to identify practices related to death notification deemed less useful by survivors (Eberwein, 2006). From what emerged, notifiers should avoid providing unsolicited advice or encouragement for a rapid recovery. During notification, they should not endorse any specific emotional attitude or attempt to identify with the survivor's experience. Instead, positive notification approaches would include: authentic expressions of closeness and concern on the part of the notifier, and the opportunity to let survivors venting their feelings while counting on the presence of another person during the notification process. Another aspect considered as important is the type of language to be used (clear, simple, and direct) and the tone of voice (which should express confidence). These are details, in fact, that a survivor could potentially remember throughout his/her life and therefore deserve special attention. On the other hand, people who are experiencing a loss first of all need respect, a fundamental prerequisite for an adequate notification of death (Eberwein, 2006).

The review also revealed that it would be important being able to predict possible emotional responses of survivors, in order to carefully select notifiers, keeping in mind the personality of each operator and their communicative and expressive styles (Adamowski et al., 1993). It has been seen, for example, that a person with high anxiety levels, with fear of death and strong apprehension related to communication, could feel strongly unable to manage a notification process and be predisposed to avoiding issues related to death (Daly and McCroskey, 1984). Indeed, it must be considered that the notification process is a highly stressful task for notifiers, both physically and emotionally (Hart and DeBernardo, 2004). On the other hand, it has been seen that staff anxiety could be reduced with a better understanding of the responses experienced by survivors at the time of notification (Finlay and Dallimore, 1991), such as denial, withdrawal, anger, sadness, isolation, and self-reported auto- or hetero guilt feelings (in the case of deaths by suicide and murder) (Wright, 1996; Constantino et al., 2002).

To date, there are still few studies describing initiatives with training programs that provide practical recommendations on who should carry out a death notification and how and when this should take place. Educational programs have been proposed to improve communication skills (Nordstrom et al., 2011), as well as the integration of death notification protocols within University courses of those students potentially involved in emergency situations (Baghcheghi et al., 2011). The GRIEV_ING Death Notification Program is among the most popular protocols; through the enhancement of specific immersive experiences, simulations and roleplaying, it aims to improve the communication skills necessary to face a death notification task within different contexts (Benenson and Pollack, 2003; Karam et al., 2017). The GRIEV_ING educational intervention is considered useful in improving the notifier's self-efficacy and the general sense of trust within the notification process. Focused on promoting the development of empathic communication, the intervention prepares notifiers to competently address emotionally charged topics, frequently associated with increased stress (Hobgood et al., 2005).

In addition to improved ability to notify, some studies have demonstrated the usefulness of these programs also in improving the self-esteem of the personnel involved in the demanding task of death notification, limiting work stress and burnout (Karam et al., 2017).

### Limitations of the Study

Our review has several limitations. First, there was no review protocol. Second, only studies written in English were taken into consideration. Third, the between-study heterogeneity was high with regards to study design, quality of study, types of death, and targets, making quite difficult to create standardized categorizations for all articles. In addition, thematic overlaps were frequent, with the majority of studies reporting general indications on all important areas of the topic, sometimes in a succinct manner, some others in a quite extended one. These limits, together with the difficulty—on some occasions—in distinguishing between guidelines and training programs, created difficulties in the interpretation of results; for example, it was not easy to derive specific indications for specific circumstances. Furthermore, the scarcity of quantitative study doesn't allow identifying the advantages of certain types of intervention compared to others.

## Conclusions

The review of the literature and the content analysis underlined the complexity of the death notification process. Numerous variables affect its impact: circumstances of death; quality of communication (verbal or non-verbal); characteristics of the context in which the notification is carried out; presence or absence of sources of support for survivors, personality characteristics of notifier and recipient; etc.

The complexities of this sensitive area reinforce the view that more research is needed, and training gaps exist within the professional paths of the figures potentially involved in this difficult task (Smith et al., 1999; Smith-Cumberland and Feldman, 2005).

Notification skills could be improved through specific educational programs, which can direct eventual notifiers toward the acquisition of communication abilities useful for the management of emergency situations. Some of the training courses and protocols that already exist, in fact, can constitute a resource for improving one's sense of self-efficacy and general confidence in dealing with emotionally charged topics and highly stressful situations (Parrish et al., 1987; Sykes, 1989; Iserson, 1999). The painful experience of having to know that a loved one unexpectedly died can be somewhat contained and alleviated by the use of structured and multidisciplinary approaches by notifiers, as a result of staff education campaigns (Adamowski et al., 1993). Raising awareness on the consequences of a bad notification process and its long-term impact appears as particularly important. Future work could focus on aspects that are still not very well-profiled, such as the definition of notification procedures taking into account situational differences, and professional and personal characteristics of the figures most frequently involved in the task.

## Author Contributions

DDL has conceived the work, coordinated the study, and written the final manuscript. AS and CA have contributed to the early stages of the research with literature searches and summary reports. JZ provided literature search and scrutiny and several paper drafts. AVG and SD have contributed to searches and to draft parts of this review. SB made the literature search and screening of papers. All authors contributed to the article and approved the submitted version.

## Conflict of Interest

The authors declare that the research was conducted in the absence of any commercial or financial relationships that could be construed as a potential conflict of interest.
