# Supplementary material for: Notification of Unexpected, Violent and Traumatic Death: A Systematic Review
Source: Front Psychol. 2020 Sep 24;11:2229. doi: 10.3389/fpsyg.2020.02229 (PMC7546769; doi:10.3389/fpsyg.2020.02229)

**Appendix**

The complete search strategy, including all the filters used, is shown below for one of the databases that provided most research data: Ebsco Psychinfo.

• Access to the Ebsco portal was made through the Proxy of the University of Padua.

• Selected as database: PsychInfo.

• The keyword 01 was inserted in quotation marks in the first search bar.

• Each researcher limited the search to the years assigned to him/her; for example, JZ initially selected the years 1991 - 1993; later she repeated the search for the years 2003 - 2005, and finally repeated the search for the period 2015 - 2017;

• No limit was selected with respect to the options: Linked Full Text; References Available; Open Access.

• Option: all results were selected in the type of resource.

• Retrieved records were copied and pasted into a Word file.

• Entered the keyword 02 in quotes in the first search bar.

• The same research methodology was repeated.

• Retrieved records were copied and pasted into a Word file.

• The keyword 03 was inserted in quotation marks in the first search bar.

• Same research methodology was repeated.

• Retrieved records were copied and pasted into a Word file.

• The keyword 04 was inserted in quotation marks in the first search bar.

• The same research methodology was repeated.

• Retrieved records were copied and pasted into a Word file.

• For keyword 05 the first term (notification) was entered in the first search bar.

• The first AND option was used by entering "traumatic death" in the search bar in quotation marks.

• The same research methodology was repeated.

• Retrieved records were copied and pasted into a Word file.

• For keyword 06 the first term (communication) was entered in the first search bar.

• The first AND option was used by entering "traumatic death" in the search bar in quotation marks.

• The same research methodology was repeated.

• Retrieved records were copied and pasted into a Word file.

• For keyword 07 the first term (notification) was entered in the first search bar.

• The first AND option was used by entering "sudden death" in the search bar in quotation marks.

• The same research methodology was repeated.

• Retrieved records were copied and pasted into a word file.

• For keyword 08 the first term (communication) was entered in the first search bar.

• The first AND option was used by entering "sudden death" in the search bar in quotation marks.

• The same research methodology was repeated.

• Retrieved records were copied and pasted into a Word file.]


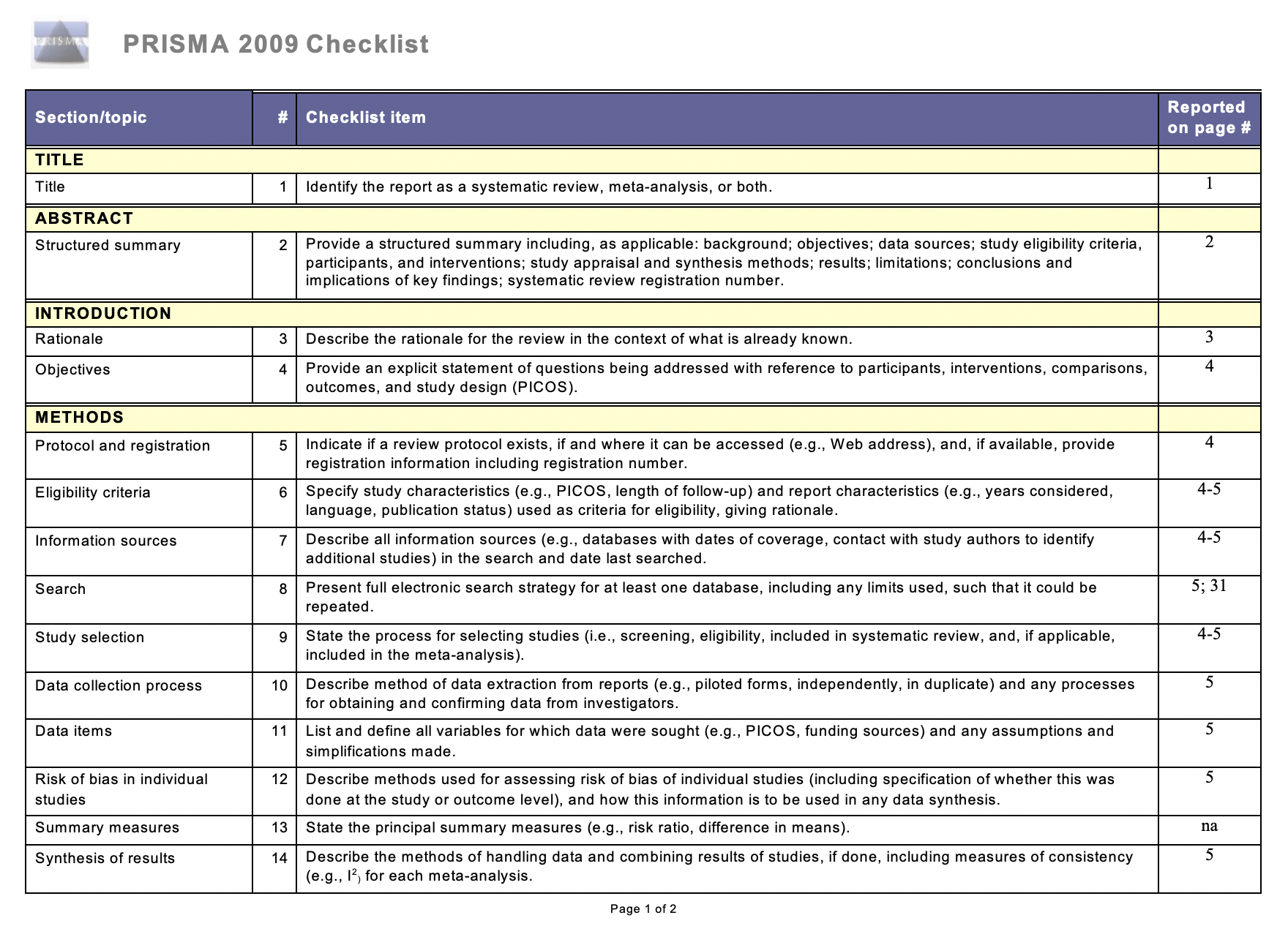


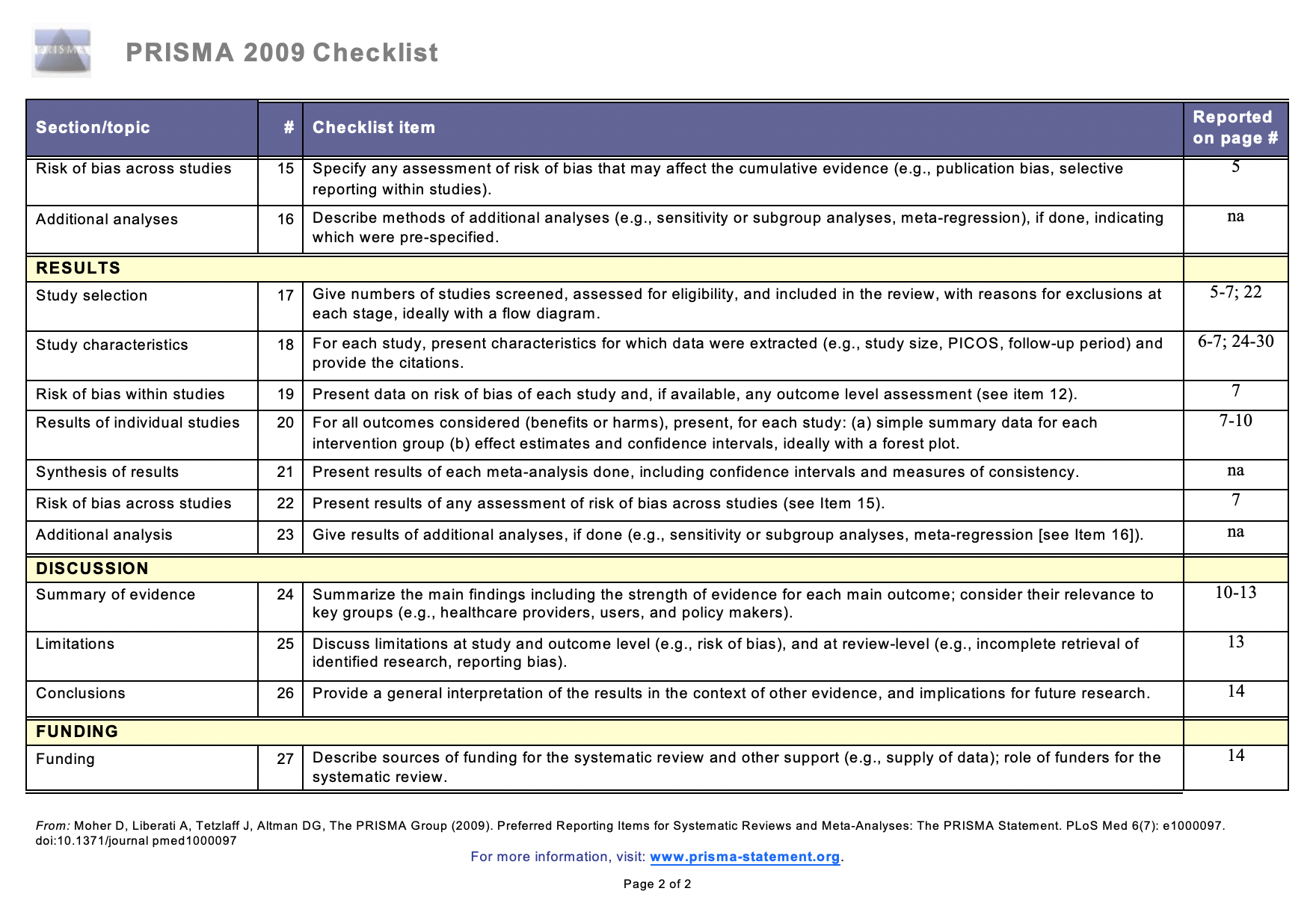

Supplement: Supplementary file 1 [file Data_Sheet_1.docx]
